# Artificial intelligence for the detection of sacroiliitis on magnetic resonance imaging in patients with axial spondyloarthritis

**DOI:** 10.3389/fimmu.2023.1278247

**Published:** 2023-11-10

**Authors:** Seulkee Lee, Uju Jeon, Ji Hyun Lee, Seonyoung Kang, Hyungjin Kim, Jaejoon Lee, Myung Jin Chung, Hoon-Suk Cha

**Affiliations:** ^1^ Department of Medicine, Samsung Medical Center, Sungkyunkwan University School of Medicine, Seoul, Republic of Korea; ^2^ Medical AI Research Center, Samsung Medical Center, Seoul, Republic of Korea; ^3^ Department of Radiology, Samsung Medical Center, Sungkyunkwan University School of Medicine, Seoul, Republic of Korea; ^4^ Department of Data Convergence and Future Medicine, Sungkyunkwan University School of Medicine, Suwon, Republic of Korea

**Keywords:** axial spondyloarthritis, MRI, artificial intelligence, machine learning, sacroiliitis

## Abstract

**Background:**

Magnetic resonance imaging (MRI) is important for the early detection of axial spondyloarthritis (axSpA). We developed an artificial intelligence (AI) model for detecting sacroiliitis in patients with axSpA using MRI.

**Methods:**

This study included MRI examinations of patients who underwent semi-coronal MRI scans of the sacroiliac joints owing to chronic back pain with short tau inversion recovery (STIR) sequences between January 2010 and December 2021. Sacroiliitis was defined as a positive MRI finding according to the ASAS classification criteria for axSpA. We developed a two-stage framework. First, the Faster R-CNN network extracted regions of interest (ROIs) to localize the sacroiliac joints. Maximum intensity projection (MIP) of three consecutive slices was used to mimic the reading of two adjacent slices. Second, the VGG-19 network determined the presence of sacroiliitis in localized ROIs. We augmented the positive dataset six-fold. The sacroiliitis classification performance was measured using the sensitivity, specificity, and area under the receiver operating characteristic curve (AUROC). The prediction models were evaluated using three-round three-fold cross-validation.

**Results:**

A total of 296 participants with 4,746 MRI slices were included in the study. Sacroiliitis was identified in 864 MRI slices of 119 participants. The mean sensitivity, specificity, and AUROC for the detection of sacroiliitis were 0.725 (95% CI, 0.705–0.745), 0.936 (95% CI, 0.924–0.947), and 0.830 (95%CI, 0.792–0.868), respectively, at the image level and 0.947 (95% CI, 0.912–0.982), 0.691 (95% CI, 0.603–0.779), and 0.816 (95% CI, 0.776–0.856), respectively, at the patient level. In the original model, without using MIP and dataset augmentation, the mean sensitivity, specificity, and AUROC were 0.517 (95% CI, 0.493–0.780), 0.944 (95% CI, 0.933–0.955), and 0.731 (95% CI, 0.681–0.780), respectively, at the image level and 0.806 (95% CI, 0.729–0.883), 0.617 (95% CI, 0.523–0.711), and 0.711 (95% CI, 0.660–0.763), respectively, at the patient level. The performance was improved by MIP techniques and data augmentation.

**Conclusion:**

An AI model was developed for the detection of sacroiliitis using MRI, compatible with the ASAS criteria for axSpA, with the potential to aid MRI application in a wider clinical setting.

## 1 Introduction

Axial spondyloarthritis (axSpA) is a chronic inflammatory disease that predominantly affects the axial skeleton, including the sacroiliac (SI) joints (1, 2). Historically, axSpA was the result of the recognition of the early phases of the disease, termed ankylosing spondylitis (AS). In the era of the modified New York criteria, SI joint damage had to be evident on plain radiographs to fulfil the criteria for AS (3). However, magnetic resonance imaging (MRI) can recognize inflammation of the SI joints before the development of erosions that show radiographic changes on plain radiographs. Therefore, MRI of the SI joints is becoming increasingly important for early diagnosis and treatment of axSpA (4). Among the several MRI findings observed in axSpA, subchondral bone marrow edema (BME), which indicates active inflammation, was identified as a finding suggestive of sacroiliitis. The Assessment of SpondyloArthritis international Society (ASAS) criterion defined the presence of definite subchondral BME that is highly suggestive of sacroiliitis in semi-coronal short tau inversion recovery (STIR) sequences as “positive MRI” (5). Despite these advances, MRI interpretation of SI joints is labor-intensive, requires the acquisition of special skills, and shows variable results, even among experienced specialists (6).

The range of AI applications has recently expanded. Image data are advantageous for learning using AI because the input is presented as objective numbers, and the amount of data is large. Therefore, AI exhibits excellent performance in the field of picture recognition (7). Based on these results, efforts have been made to apply AI to various medical imaging (8, 9). In patients with axSpA, studies have used plain radiographs as inputs for machine learning to detect radiographic sacroiliitis (10) or the extent of radiographic progression (11).

Deep learning is a subtype of AI that uses many hidden layers for nonlinear process and extraction of important features. Deep learning has previously been applied to various MRI data (12–14) including that of musculoskeletal system (15, 16). Therefore, the use of deep learning to detect sacroiliitis on the MRI of patients with axSpA appears promising. The Faster Region-based Convolutional Neural Network (R-CNN) (17) and Visual Geometry Group (VGG) network (18) are types of deep learning that have shown high performance in image classification tasks, owing to their computational efficacy (19) and higher performance than traditional methods (19–22).

In this study, we used Faster R-CNN and VGG-19 to detect sacroiliitis according to the ASAS definition of positive MRI in patients with axSpA.

## 2 Materials and methods

### 2.1 Study sample

This retrospectisve study was conducted at the Samsung Medical Center, a tertiary referral hospital in Seoul, South Korea. We included patients who (1) visited the rheumatology clinic because of chronic back pain (> 3 months), (2) underwent semi-coronal MRI scans of the SI joints with STIR sequences between January 2010 and December 2021, and (3) were older than 18 years. We excluded cases in which the inflammation of the SI joint could not be evaluated because of artifacts, such as in patients who underwent total hip replacement, and patients with inconclusive clinical diagnoses. The STIR sequences of the SI joints were obtained using four 3.0-T MRI scanners from two companies: Ingenia, Ingenia CX, and Achieva from Phillips Healthcare and Skyra from Siemens Healthineers for 47, 26, 80, and 143 patients, respectively.

### 2.2 Data labeling

Sacroiliitis was identified independently by a rheumatologist (SL) and radiologist (JHL) using the definition of MRI sacroiliitis according to the ASAS classification criteria for axSpA. Active inflammatory lesions of the SI joint associated with spondyloarthritis on MRI were defined as the presence of BME visualized as hyperintensity in at least two consecutive slices or in at least two locations within a single slice, according to the ASAS criteria (5). The raters were blinded to the clinical data. Discrepancies in interpretation were resolved by a consensus. In addition, we extracted the bilateral SI joints by drawing bounding boxes to reduce the noise of unnecessary information when evaluating sacroiliitis. These ROIs were independently outlined by a rheumatologist (SL) and a radiologist (MCJ).

### 2.3 Model training

**Figure 1:**
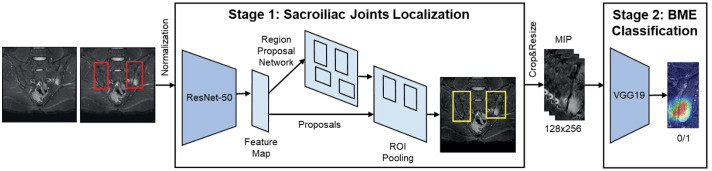
Artificial intelligence framework to detect sacroiliitis in accordance with the assessment of spondyloArthritis international society criteria for axial spondyloarthritis.

The pipeline for the proposed automatic sacroiliitis classification method is shown in **Figure 1**. First, all MR images were normalized, and the ROIs of the SI joints were extracted from the entire MRI. The presence of sacroiliitis was determined on individual MRI slices of localized SI joint ROIs.

### 2.4 Image pre-processing and sacroiliac joints localization

MRI shows a large intensity variation between different patients, as well as different slices within one patient (**Supplementary Figure 1**). Thus, we applied adaptive histogram equalization (23) to three-dimensional volume MRI of each patient to normalize the MRI for each patient. The regions other than SI joints can affect the diagnosis of sacroiliitis and interfere in the extraction of important features for the diagnosis of sacroiliitis (24). Thus, a deep convolutional neural network was used to efficiently localize the SI joints and automatically extract the ROIs. First, Faster R-CNN with ResNet-50 (25) was used to extract the ROIs of the SI joints using the entire MRI (**Supplementary Figure 2A**). Second, the ROIs extracted by the network were cropped and resized to a resolution of 128 × 256 for use as inputs to the classification network (**Supplementary Figure 2B**). One ROI each was extracted from the left and right sides of the SI joint.

### 2.5 Image post-processing and sacroiliitis classification

The classification network determined the presence of sacroiliitis based on the brightness distribution of pixels in the localized ROIs and contextual information based on the positional relationships between consecutive slices. MIP (26) was applied to three consecutive slices to mimic the process of comparing two adjacent slices before identifying the inflammatory lesion. In addition, the class imbalance between the positive and negative labels causes the network to become overfit for the majority class (negative labels). To overcome this problem, we utilized data augmentation techniques, including blurring, adjusting contrast, adding noise, rotating, and sharpening of positive labels. Because acquiring a large number of MRI scans for the diagnosis of sacroiliitis is difficult, we used transfer learning (27) to effectively train the network. As shown in **Figure 1**, the pre-trained VGG-19 was applied as a classification network using post-processed localized ROIs as inputs. The architecture of VGG-19 used in this study is illustrated in **Supplementary Figure 3**. More details are provided in the **supplemental methods**.

### 2.6 Comparing prediction results with ground truth of sacroiliitis and clinical diagnosis

Through each one-round cross-validation, each individual obtained a prediction result once. Because we performed a three-round cross-validation, each individual in this study had three prediction results. We repeated the prediction thrice to robustly compare the prediction performance by randomly dividing the training and validation groups thrice during cross-validation, which was not intended to confirm individual prediction results. However, to compare the ground truth and prediction results, we defined a prediction as positive when a patient was unanimously predicted to have sacroiliitis by all three predictions. We then compared the prediction results with the ground truth used for the labeling and diagnosis of axSpA by a rheumatologist based on a combination of clinical factors. We defined the patients who were clinically diagnosed with axSpA as ‘axSpA’ group, and the patients who were not diagnosed with axSpA as ‘nonspecific back pain’ group.

### 2.7 Statistical analyses

To evaluate the sacroiliitis classification performance, we compared the proposed method (Method C) with sacroiliitis classification results without augmentation and MIP (Method A) and sacroiliitis classification results using augmentation but without MIP (Method B). Sensitivity, specificity, and AUROC were used to determine the performances of classification algorithms. The intersection over union (IoU) was used to measure the gap between the ground truth and the predicted results of ROIs. The performances were obtained by averaging nine performances from three-round three-fold cross-validation. When assessing the performance of the AI model using the test dataset, we refrained from employing data augmentation and instead made predictions using the trained model with real MRI images. This approach was chosen to evaluate performance under actual conditions, without the use of simulated input images. More details are provided in the eMethods.

Statistical analyses of the clinical variables were performed using R version 4.1.3 (28). The interobserver reliability of positive sacroiliitis on MRI was determined using Cohen’s kappa coefficient (29). The chi-square test was performed to compare the prediction results of the individual patients with the ground truth and the clinical diagnosis of axSpA. All p-values were two-sided, and a p-value less than 0.05 was considered statistically significant.

## 3 Results

### 3.1 Characteristics of participants

A total of 296 participants with 4,746 MRI slices were included in this study (**Supplementary Figure 4**). A total of 167 participants had axSpA and 129 had nonspecific back pain. Sacroiliitis consistent with axSpA, as defined by the ASAS classification criteria, was identified in 119 participants (**Supplementary Table 1**). The clinical characteristics of the participants who underwent MRI are presented in **Table 1**. The participants were 36.8 years old on average and predominantly male (174/296, 58.8%). Among the participants with axSpA, 96 (57.5%) fulfilled the modified New York radiographic criteria for AS, and 153 (91.6%) were human leukocyte antigen B27 positive (153/167, 91.6%). The mean C-reactive protein level in patients with axSpA was 1.37 mg/dl.

**Table 1:** Baseline characteristics of patients.

	All patients (n=296)	axSpA (n=167)	Nonspecific chronic back pain (n=129)	p-value
Age, mean (SD), years	36.78 (12.45)	35.78 (12.63)	38.07 (12.14)	0.117
Male sex (%)	174 (58.8)	104 (62.3)	70 (54.3)	0.204
Duration of backpain, mean (SD), months	38.07 (51.57)	46.58 (59.13)	26.45 (36.12)	0.002
Inflammatory back pain (%)	183 (61.8)	127 (76.0)	56 (43.4)	<0.001
Peripheral arthritis (%)	68 (23.0)	45 (26.9)	23 (17.8)	0.087
Enthesitis (%)	32 (10.8)	21 (12.6)	11 (8.5)	0.356
Dactylitis (%)	3 (1.0)	2 (1.2)	1 (0.8)	>0.999
IBD (%)	6 (2.0)	3 (1.8)	3 (2.3)	>0.999
Uveitis (%)	49 (16.6)	36 (21.6)	13 (10.1)	0.013
Psoriasis (%)	6 (2.0)	1 (0.6)	5 (3.9)	0.117
HLA-B27 positivity (%, n=288)	205 (69.3)	153 (91.6)	52 (40.3)	<0.001
Negative	83 (28.0)	9 (5.4)	74 (57.4)	
Positive	205 (69.3)	153 (91.6)	52 (40.3)	
Unknown	8 (2.7)	5 (3.0)	3 (2.3)	
ESR, mean (SD), mm/h	25.63 (29.06)	33.37 (33.68)	15.78 (17.53)	<0.001
CRP, mean (SD), mg/dL	0.92 (2.03)	1.37 (2.44)	0.34 (1.08)	<0.001
ASAS axSpA criteria (%)		161 (96.4)		
Non-radiographic axSpA (%)		71 (42.5)		
NSAIDs (%)		136 (81.4)		
Sulfasalazine (%)		51 (30.5)		
Other csDMARDs (%)^a^		5 (3.0)		
Biologics (%)		12 (7.2)		
Any kind of treatment for axSpA (%)		140 (83.8)		

IBD, inflammatory bowel disease; HLA-B27, human leukocyte antigen B27; ESR, erythrocyte sedimentation rate; CRP, C-reactive protein; ASAS, Assessment of SpondyloArthritis international Society; axSpA, axial spondyloarthritis; NSAIDs, nonsteroidal anti-inflammatory drugs; csDMARDs, conventional synthetic disease-modifying antirheumatic drugs.

a csDMARDs except sulfasalazine.

### 3.2 Interobserver reliability of sacroiliitis in MRI

The raters reached substantial agreement. The Cohen’s Kappa coefficient was 0.876 (95% CI: 0.771–0.981) for the identification of sacroiliitis compatible with the ASAS definition between the two readers (SL and JHL).

### 3.3 Localization of SI joints

First, the SI joints were localized using AI (**Supplementary Figure 5**). The average IoUs of the predicted results were 74.23% and 74.37% for the right and left SI joints, respectively (**Supplementary Table 2**). The predicted ROIs covered most SI joints, even in relatively poorly predicted cases (**Supplementary Figure 5B**). Therefore, we concluded that the AI that predicted the SI joint had sufficient performance for further analysis.

### 3.4 Detection of sacroiliitis compatible with the ASAS definition using artificial intelligence

**Figure 2:**
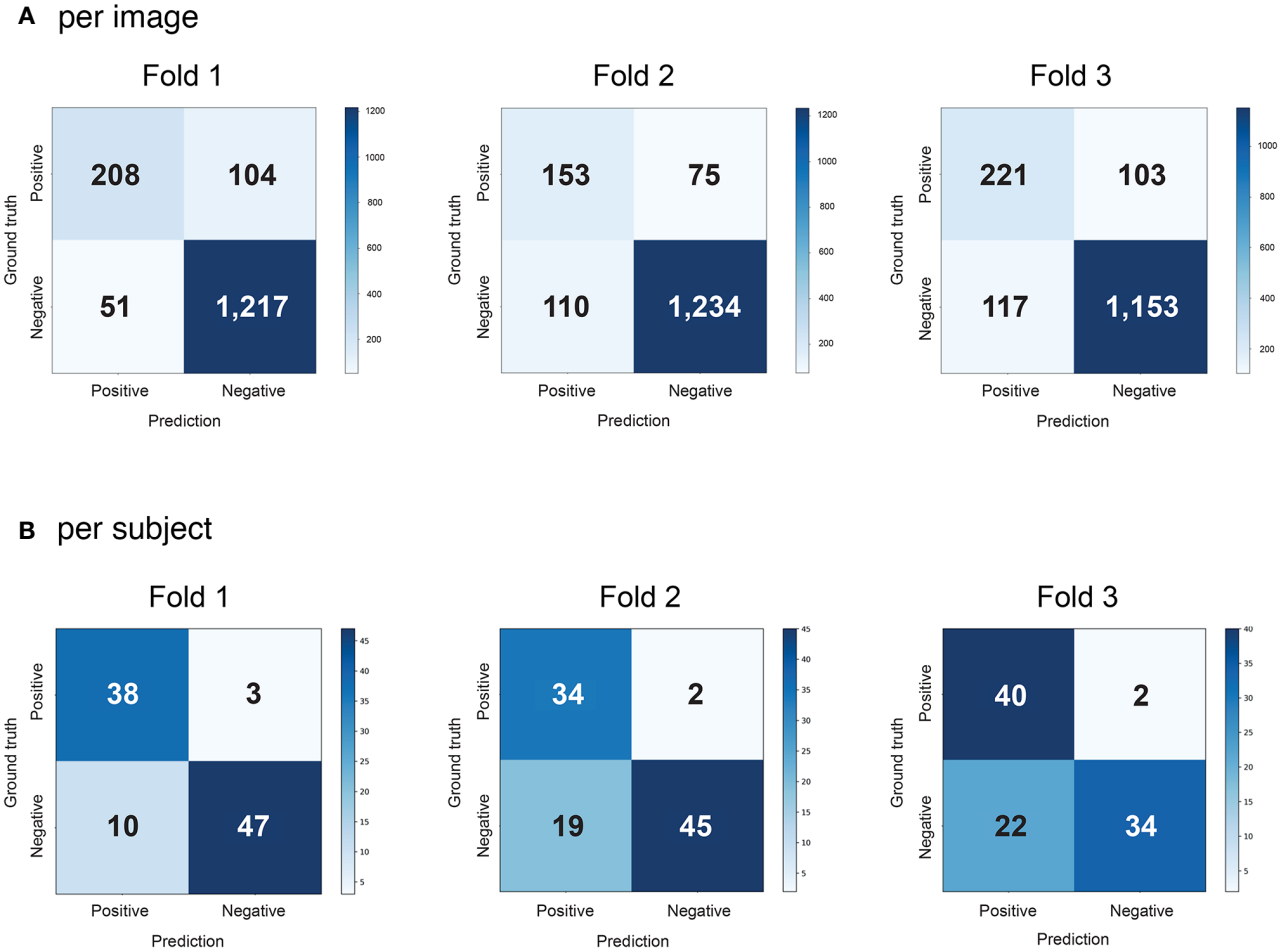
Confusion matrices of the first-round cross-validation using the proposed method (Method C) for detecting sacroiliitis **(A)** for individual MRI slices; **(B)** for each subject.

On average, the final AI model (Method C) for the detection of sacroiliitis compatible with the ASAS definition of axSpA showed a sensitivity of 0.725 (95% CI, 0.705–0.745), specificity of 0.936 (95% CI, 0.924–0.947), and AUROC of 0.830 (95% CI, 0.792–0.868) in individual MRI slices, and a sensitivity of 0.947 (95% CI, 0.912–0.982), specificity of 0.691 (95% CI, 0.603–0.779), and AUROC of 0.816 (95% CI, 0.776–0.856) in individual participants after three-round three-fold cross-validation compared with the reference standard by two raters (**Table 2**). The performance of sacroiliitis detection improved gradually from deep learning directly on the raw image (Method A) by implementing augmentation (Method B) and further by performing both augmentation and MIP (Method C). The confusion matrices for the detection of sacroiliitis per image and subject are shown in **Figure 2** and **Supplementary Figure 6**.

**Table 2:** Performances of artificial intelligence models for the detection of sacroiliitis compatible with the assessment of spondyloArthritis international society definition.

Performances by individual MRI slices			
	Sensitivity (95% CI)	Specificity (95% CI)	AUROC (95% CI)
Method A^a^	0.517 (0.493–0.780)	0.944 (0.933–0.955)	0.731 (0.681–0.780)
Method B^b^	0.563 (0.538–0.587)	0.933 (0.920–0.945)	0.747 (0.699–0.796)
Method C^c^	0.725 (0.705–0.745)	0.936 (0.924–0.947)	0.830 (0.792–0.868)
Performances by each patient			
	Sensitivity	Specificity	AUROC
Method A^a^	0.806 (0.729–0.883)	0.617 (0.523–0.711)	0.711 (0.660–0.763)
Method B^b^	0.859 (0.792–0.926)	0.589 (0.494–0.684)	0.722 (0.671–0.774)
Method C^c^	0.947 (0.912–0.982)	0.691 (0.603–0.779)	0.816 (0.776–0.856)

AUROC, area under the receiver operating characteristic curve.

a Artificial intelligence model for the detection of sacroiliitis without augmentation and maximum intensity projection.

b Artificial intelligence model for the detection of sacroiliitis using augmentation without maximum intensity projection.

c Artificial intelligence model for the detection of sacroiliitis using both augmentation and maximum intensity projection.

### 3.5 Comparing prediction results with the ground truth of sacroiliitis and clinical diagnosis

We compared the prediction results with the ground truth of sacroiliitis and the clinical diagnosis of axSpA (**Supplementary Table 3**). A total of 21 false-positive cases occurred in the prediction by the unanimous decision of the predictive value of the three rounds. Of the 21 false-positive cases, 10 occurred in patients who were not clinically diagnosed with axSpA and 11 in patients who were clinically diagnosed with axSpA. A total of 53 patients existed who did not meet the ASAS criteria for “positive MRI” but were clinically diagnosed with axSpA, and the model generated in this study showed that significantly more false positives occurred in patients with clinically diagnosed axSpA than in those without (p=0.033, **Supplementary Table 4**). On the other hand, because patients with negative clinical diagnoses of axSpA had only positive sacroiliitis in five patients, we could not find additive information comparing false-negative cases with clinical diagnoses.

**Figure 3:**
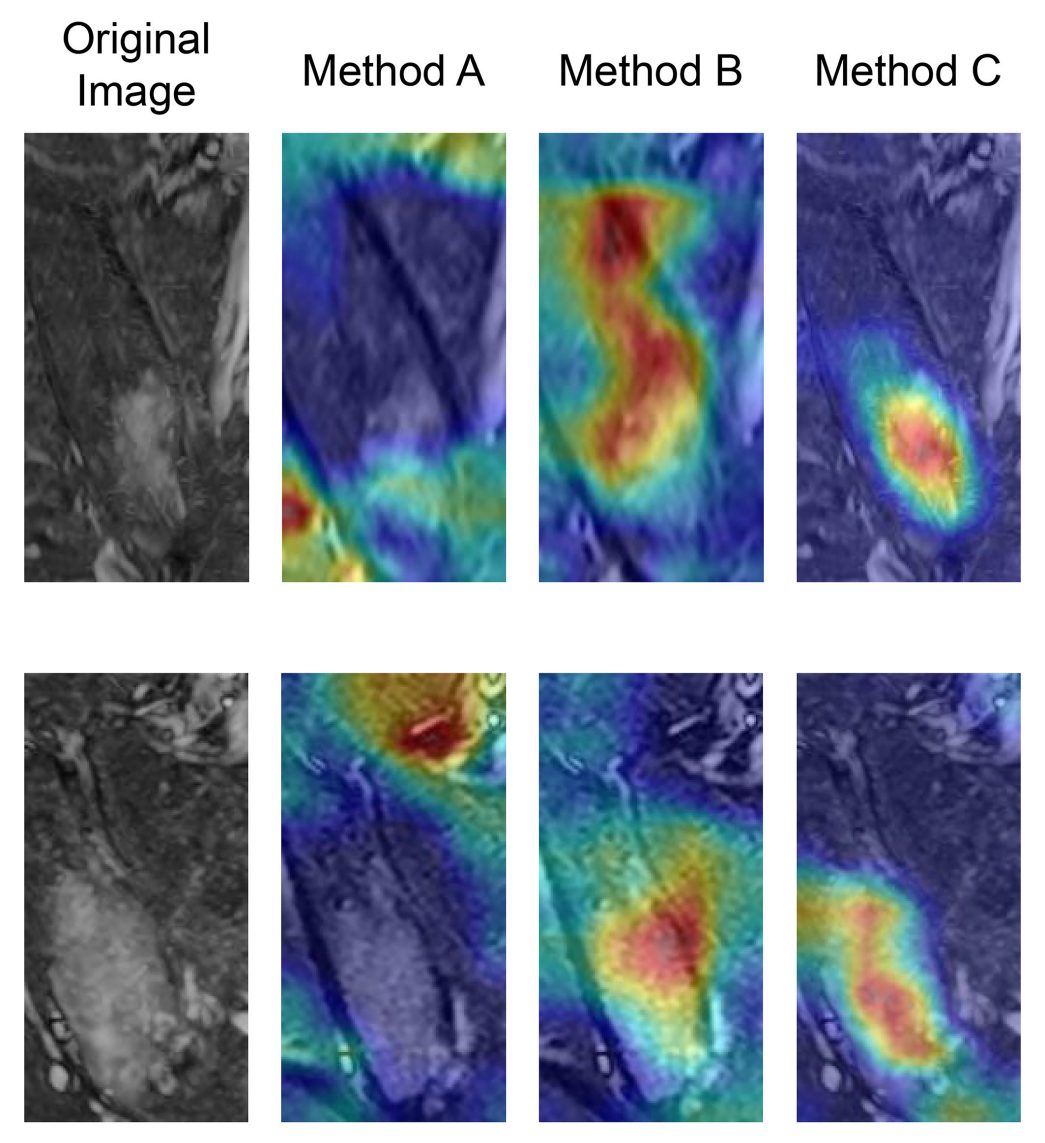
Examples of gradient-weighted class activation mapping (Grad-CAM) for the classification model from two different patients. The place of bone marrow edema matched with higher activations of Grad-CAM in method C, better than method A or B.

**Figure 3** shows examples of gradient-weighted class activation mapping (Grad-CAM) used to visualize the models’ decisions and highlight the regions relevant to model predictions. In these examples, the place of BME matched with higher activations of Grad-CAM in method C, better than method A or B.

## 4 Discussion

We generated AI models to detect sacroiliitis according to the ASAS definition of positive MRI in patients with axSpA. We used the Faster R-CNN and VGG-19 algorithms, and the performance was improved by MIP techniques and data augmentation. The AUROCs were above 0.8 on an individual slice basis and on a per-patient basis.

MRI has recently gained importance in the diagnosis of axSpA and the assessment of disease activity because it can detect inflammation in patients with axSpA before structural changes are observed on plain radiographs. However, MRI findings of sacroiliitis in the ASAS criteria may have false positives (30) or false negatives (31). Therefore, MRI readings for sacroiliitis require a specialist with experience in MRI readings of musculoskeletal diseases.

This study included all MRI scans to evaluate the SI joint that were performed over a 12-year period in the rheumatology department for chronic back pain. This allowed the inclusion of a diverse patient population, including patients with nonspecific back pain and axSpA. An AI model was created and evaluated using MRI scans from four different machines of two different companies. Therefore, this study had the advantage of including all MRI scans performed to evaluate sacroiliitis in real-world clinical practice, allowing us to evaluate the model in the same context as patients encountered in a real-world clinical practice.

Notably, increasing the positive dataset through augmentation and training with MIP to include data from the anterior and posterior image slices improved the performance of the AI model. When collecting data to determine the presence of sacroiliitis, a small number of image slices contained actual inflammatory lesions, even in patients with positive axSpA MRI findings. Because patients without sacroiliitis must also be included in the training, the negative dataset will always outnumber the positive dataset for learning about active inflammatory sacroiliitis. In this case, augmentation increased the number of positive datasets. In addition, because the definition of positive MRI in the ASAS criteria for axSpA includes cases in which a single BME is observed in two or more consecutive image slices, to implement this, we used MIP to consider the anterior and posterior slices together in training. Because we confirmed that the augmentation and MIP techniques applied with a theoretical background improved the performance of the AI model, we expect that the same process will improve the performance of AI models with different structures in the future.

Several studies have examined active inflammation in SI joints using machine learning (10, 24, 32). Previous studies had comparable performance in predicting active inflammatory lesions in SI joints, and the definition of active inflammation was based on positive MRI findings compatible with the ASAS criteria for axSpA, as in the present study. However, no study has compared predicted results with the clinical diagnosis of axSpA.

The inclusion of MRI in radiographic diagnosis in the ASAS criteria has greatly improved the early diagnosis of axSpA. However, with only active inflammation included in the criteria, concerns exist about false positives and false negatives. As already known, BME can be observed on MRI as a false positive in osteitis condensans ilii or athletes with high physical activity (30, 33, 34), and in chronic disease, active inflammation may not be observed and may be reported as a false negative (31). In this study, patients without active inflammation based on the ASAS criteria for axSpA, but with a positive AI prediction, were significantly more likely to be clinically diagnosed with axSpA. Although the exact factors that contributed to this could not be analyzed in this study, probably, factors other than active inflammation were trained together and contributed to the prediction of clinical axSpA. Therefore, in addition to active inflammation, other factors around the SI joint might contribute to the diagnosis of axSpA in our AI model.

A few types of chronic inflammatory lesions have also been described as specific MRI findings of axSpA. Sclerosis, erosion, fat deposition, and ankylosis are typical of axSpA (5), and these findings are significantly less common in athletes or postpartum conditions which may show BME similar to axSpA (34, 35). However, quantitative criteria for the classification of axSpA for chronic inflammatory lesions have not yet been established and are not included in the ASAS criteria for the definition of positive MRI. Furthermore, T1-weighted images are required for evaluation in addition to the STIR sequences used in this study. For this reason, this analysis was not performed in this study, but it is expected that chronic inflammatory lesions will play a role in improving prediction performance.

In contrast, previous research suggested an association between the presence of non-inflammatory spine abnormalities and BME, fulfilling the ASAS definition of MRI sacroiliitis in patients with definite mechanical chronic back pain (36). Based on this, it is expected that by considering non-inflammatory bony abnormalities together, we may achieve a better differentiation of sacroiliitis caused by mechanical issues. Additionally, we believe that including images of the spine, another major site commonly affected by axSpA, would be advantageous for the predictive model. However, because accurate labeling is required for the creation of a prediction model, we still need to evaluate the clinical utility of non-inflammatory lesions and spine images for the diagnosis of axSpA applying them to a prediction model for sacroiliitis. Our study had several limitations. First, because no true ground truth existed for the presence of axSpA sacroiliitis, the consensus of multiple experts was used as the ground truth. The diagnosis uncertainty introduces noise into the dataset and affects the performance of the model. However, we assumed that the ground truth was of good quality because the two experts agreed substantially, with high interobserver reliability. Second, this study was designed to detect active inflammation; therefore, it could not distinguish between BME caused by other factors, such as physical activity or childbirth. These changes might be difficult to distinguish from axSpA based on imaging findings alone. Third, the AI model could not be evaluated using MRI scans from different institutes. Therefore, generalizing these results to MRI images is difficult using new scanners and protocols. However, because the study was trained and tested using images acquired from four different MRI machines over a 12-year period, we believe that it can be applied to MRI images captured in new environments. Fourth, one of the conditions for positive MRI to qualify for the ASAS criteria, finding at least two independent BME in a single slice, was not included in the model generation. The ASAS criteria mention two conditions regarding the amount of signal required to determine a positive MRI: if there is only one signal, it should be present in at least two slices (5). If there is more than one signal in a single slice, one slice may be sufficient. The MIP method was introduced for training when a single signal was identified in at least two consecutive slices; however, the method for identifying the existence of more than two independent signals in one slice was not implemented separately when creating the model. Although we did not provide information regarding the presence of two independent signals, we believe that additional post-processing may not be required because all the information is already in a single given, and previous studies have also achieved successful results without the information needed to detect distinct BMEs in a single slice (10, 32). Fifth, we did not test the AI model in practical applications. Although we believe that the AI model significantly reduces inter-observer variability and is useful for identifying sacroiliitis in a practice setting without a musculoskeletal imaging specialist, our study was not tested in a real-world setting. Further research is required to demonstrate the usefulness of the AI model in real-world practice.

In conclusion, an AI model was developed for the detection of sacroiliitis on MRI, compatible with the ASAS criteria for axSpA, with the potential to aid MRI applications in a wider clinical setting.

## Data availability statement

The original contributions presented in the study are included in the article/**Supplementary Material**. Further inquiries can be directed to the corresponding author.

## Ethics statement

This study complied with the principles of the Declaration of Helsinki. The Institutional Review Board of the Samsung Medical Center approved this study (SMC 2021-10-121). This study was performed using the data extracted from the Clinical Data Warehouse (CDW) Darwin-C at the Samsung Medical Center. The requirement for informed consent was waived because we used only de-identified data collected from the CDW.

## Author contributions

SL: Conceptualization, Data curation, Formal Analysis, Funding acquisition, Investigation, Methodology, Resources, Validation, Writing – original draft, Writing – review and editing. UJ: Conceptualization, Data curation, Formal Analysis, Investigation, Methodology, Software, Validation, Visualization, Writing – original draft, Writing – review and editing. JHL: Conceptualization, Formal Analysis, Investigation, Writing – review and editing. SK: Conceptualization, Writing – review and editing. HK: Conceptualization, Writing – review and editing. JL: Conceptualization, Writing – review and editing. MC: Conceptualization, Formal Analysis, Funding acquisition, Investigation, Methodology, Resources, Software, Supervision, Writing – review and editing. H-SC: Conceptualization, Resources, Supervision, Writing – review and editing.
