# Supplementary material for: Artificial intelligence for the detection of sacroiliitis on magnetic resonance imaging in patients with axial spondyloarthritis
Source: Front Immunol. 2023 Nov 10;14:1278247. doi: 10.3389/fimmu.2023.1278247 (PMC10676202; doi:10.3389/fimmu.2023.1278247)
Supplement: Supplementary file 1 [file DataSheet_1.docx]

Supplementary Material

**Supplementary methods**

**Machine Learning Details**

All experiments were conducted on a machine with an Intel(R) Core(tm) i7-10700K, CPU@3.8 GHz, RAM 96 GB, and NVIDIA Geforce RTX3090 Ti GPU. The PyTorch framework, PyCharm software, and Anaconda Python 3.8 interpreter built the network framework. We trained the Faster R-CNN model for 500 epochs with a cross-entropy loss function, batch size of four, and an SGD optimizer with a learning rate of 0.00001. We trained the VGG-19 model for 100 epochs with a cross-entropy loss function, batch size of four, and an Adam optimizer with a learning rate of 0.000001.

**Performance Evaluation Details**

For qualitative evaluation, the results of sacroiliac joint localization and sacroilitis classification were compared with the ground truth. For a quantitative evaluation, the intersection over union (IoU), sensitivity, specificity, and the area under the receiver operating characteristic curve (AUROC) were used to measure the gap between the ground truth and the predicted results used in the comparison and proposed methods, as shown in Equation (1).

| $IoU=\frac{TP}{TP+FN+FP}\times100$ |  |
| --- | --- |
| $Sensitivity=\frac{TP}{TP+FN}$ |  |
| $Specificity=\frac{TN}{TN+FP}$ | (1) |
| $AUC=1-\frac{1}{2}(\frac{FP}{FP+TN}+\frac{FN}{FN+TP})\times100$ |  |

In this equation, true positive (TP) and true negative (TN) denote the cases in which the predictive and ground truth values are identical, and false positive (FP) and false negative (FN) denote the cases in which the predictive and ground truth values are different. Quantitative evaluation performances were obtained by performing three-fold cross-validation thrice.

**Supplementary Figures**

**Supplementary Figure 1.** Intensity variation in MRI images: (A) Intensity variation between different patients; (B) Intensity variation between slices within one patient.

**Supplementary Figure 2.** Automatic localization of the sacroiliac joints: (A) Sacroiliac joint localization; (B) After cropping and resizing the regions of interest. (Red: ground truth; Yellow: predicted bounding box).

**Supplementary Figure 3.** Architecture of the pretrained VGG-19. The output layer is designed to generate a binary output, either positive or negative.


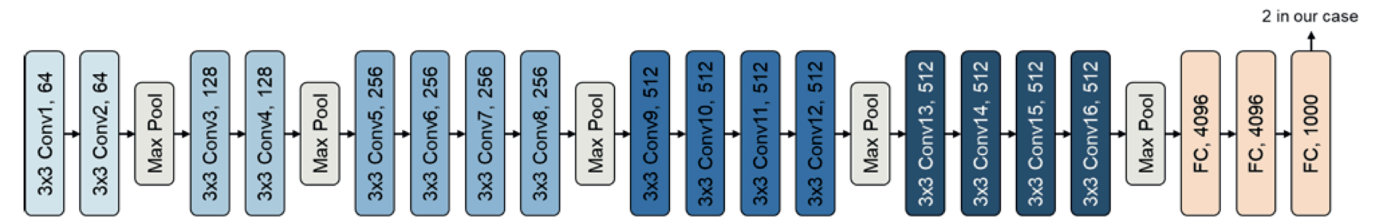


**Supplementary Figure 4.** Flowchart for the participant population**.**

**
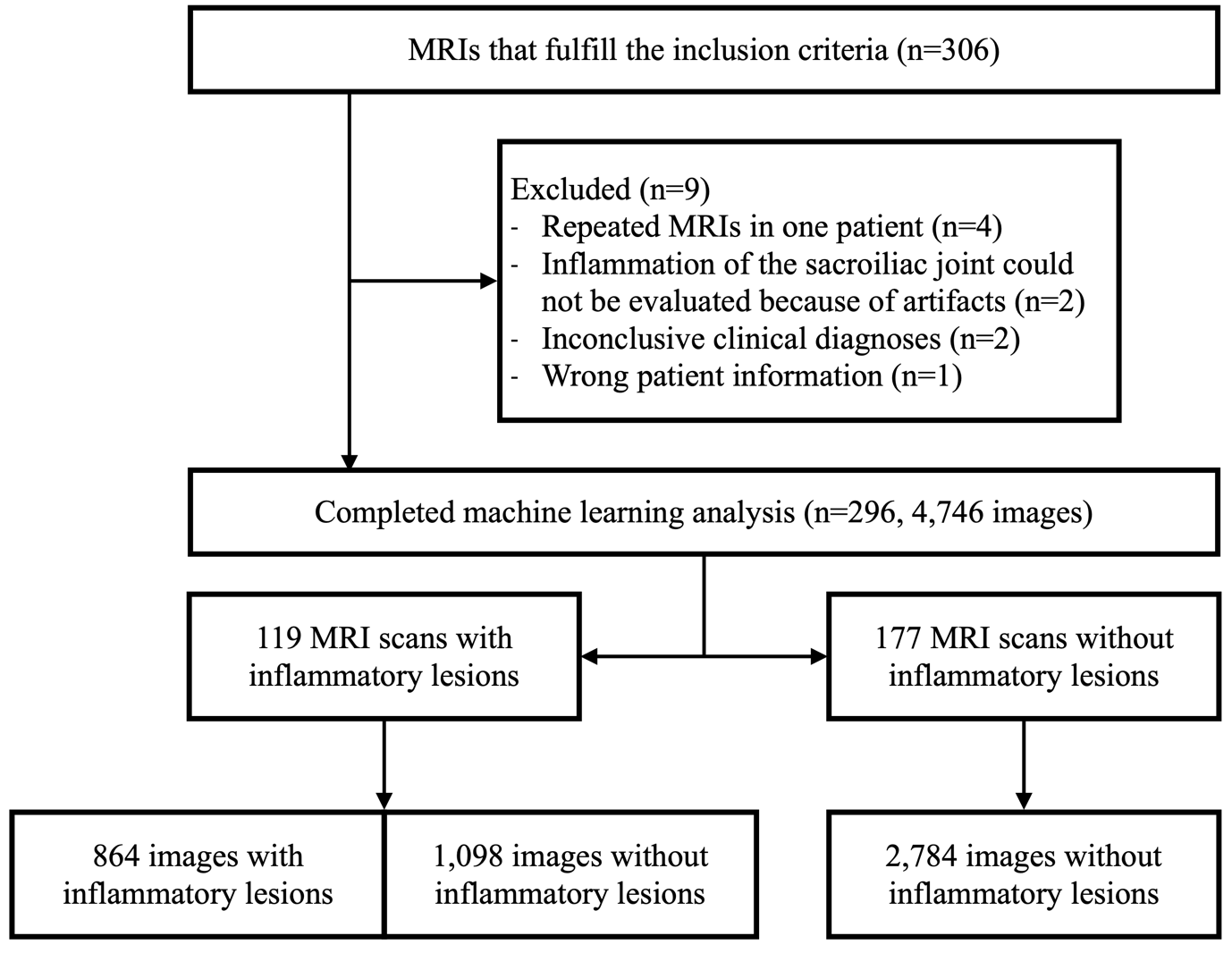
**

**Supplementary Figure 5.** Localization results of sacroiliac joints in the first step of the artificial intelligence model. (A) Examples of well-predicted regions of interest. (B) Examples of poorly predicted regions of interest. (Red: ground truth; Yellow: predicted bounding box).

**Supplementary Figure 6.** Confusion matrices of the second and third rounds of cross-validation using the proposed method (Method C) for detecting sacroiliitis: (A) second round for individual MRI slices; (B) second round for each subject; (C) third round for individual MRI slices; (D) third round for each subject.

**Supplementary Tables**

**Supplementary Table 1. Classification of radiographic findings in cases with a positive MRI according to** the Assessment of SpondyloArthritis International Society (ASAS) criteria for axial spondyloarthritis.

|  | **No. of patients (%)**  **(positive MRI, total n = 119)** |
| --- | --- |
| One BME per MRI slice with at least two consecutive slices | 90 (75.6) |
| More than one BME on a single slice | 117 (98.3) |
| Satisfying both criteria | 92 (77.3) |

MRI, magnetic resonance imaging; BME, bone marrow edema.

**Supplementary Table 2.** Prediction performances of sacroiliac joints.

|  | **First round** | **Second round** | **Third round** | **Average** |
| --- | --- | --- | --- | --- |
| Average IoU of right SI joints | 74.98 | 73.79 | 73.93 | 74.23 |
| Average IoU of left SI joints | 75.22 | 73.66 | 74.26 | 74.37 |

IoU, Intersection over Union; SI, sacroiliac.

**Supplementary Table 3.** Comparison of prediction results with ground truth for sacroiliitis and the clinical diagnosis of axial spondyloarthritis.

| Comparing the ground truth of sacroiliitis with the clinical diagnosis of axSpA | | |
| --- | --- | --- |
| Clinical diagnosis of axSpA | Ground truth for sacroiliitis | |
|  | Positive | Negative |
| Positive | 114 | 53 |
| Negative | 5 | 124 |
| Comparing prediction results with the ground truth of sacroiliitis | | |
| Ground truth for sacroiliitis | Prediction results for sacroiliitis | |
|  | Positive | Negative |
| Positive | 104 | 15 |
| Negative | 21 | 156 |
| Comparing prediction results with the clinical diagnosis of axSpA | | |
| Clinical diagnosis of axSpA | Prediction results for sacroiliitis | |
|  | Positive | Negative |
| Positive | 110 | 57 |
| Negative | 15 | 114 |

**Supplementary Table 4.** Comparing the number of false positives based on clinical diagnosis.

| Comparing prediction results with the clinical diagnosis of axSpA in patients with no active inflammatory sacroiliitis | | |
| --- | --- | --- |
| Clinical diagnosis of axSpA | Prediction results for sacroiliitis | |
|  | Positive (false positive) | Negative |
| Positive | 11 | 42 |
| Negative | 10 | 114 |
